# 

**Published:** 1912-05

**Authors:** 


THERE is a psychic relation between love and murder, between love and cruelty, between love and the religious emotion, between love and music and art and science, a fact, startling within itself. Further, these emotions or impulses are, in certain individuals, inter-convertible, like the various forms of energy in the domain of physics, or co-existent in the same individual, and one may stand for the equivalent of the other; the libido may find satisfaction, vicariously, at least to some extent, in the expression, gratification and vent of the associated emotion or impulse. This is a fact well established. It is more than a coincidence that most religious conversions occur about the time of puberty in both sexes, when the sex nature is developing and is strong, and repression necessary and enforced. We have to accept the psychic relation, empirically, as a fact, without attempting an explanation of it.

—Daniel.

